# Formic acid, an organic acid food preservative, induces viable-but-non-culturable state, and triggers new Antimicrobial Resistance traits in *Acinetobacter baumannii* and *Klebsiella pneumoniae*

**DOI:** 10.3389/fmicb.2022.966207

**Published:** 2022-11-24

**Authors:** Manisha Yadav, Samridhi Dhyani, Pooja Joshi, Sakshi Awasthi, Subhash Tanwar, Vishal Gupta, Deepak K. Rathore, Susmita Chaudhuri

**Affiliations:** Department of Multidisciplinary Clinical and Translational Research, Translational Health Science and Technology Institute, Faridabad, Haryana, India

**Keywords:** VBNC, resuscitation, formic acid, food preservative, *Acinetobacter baumannii*, *Klebsiella pneumoniae*, antimicrobial susceptibility, AMR

## Abstract

Numerous human pathogens, especially Gram-negative bacteria, are able to enter the viable-but-non-culturable (VBNC) state when they are exposed to environmental stressors and pose the risk of being resuscitated and causing infection after the removal of the trigger. Widely used food preservatives like weak organic acids are potential VBNC inducers in food processing and packaging facilities but have only been reported for food-borne pathogens. In the present study, it is demonstrated for the first time that one such agent, formic acid (FA), can induce a VBNC state at food processing, storage, and distribution temperatures (4, 25, and 37^°^C) with a varied time of treatment (days 4–10) in pathogenic Gram-negative bacteria *Acinetobacter baumannii* and *Klebsiella pneumoniae*. The use of hospital-associated pathogens is critical based on the earlier reports that demonstrated the presence of these bacteria in hospital kitchens and commonly consumed foods. VBNC induction was validated by multiple parameters, e.g., non-culturability, metabolic activity as energy production, respiratory markers, and membrane integrity. Furthermore, it was demonstrated that the removal of FA was able to resuscitate VBNC with an increased expression of multiple virulence and Antimicrobial Resistance (AMR) genes in both *pathogens*. Since food additives/preservatives are significantly used in most food manufacturing facilities supplying to hospitals, contamination of these packaged foods with pathogenic bacteria and the consequence of exposure to food additives emerge as pertinent issues for infection control, and control of antimicrobial resistance in the hospital setting.

## Introduction

The notion of “viable but non-culturable” bacteria (VBNC) was first construed in 1982 when it was discovered that bacteria with persistent metabolic activity and the capacity to reproduce under certain circumstances still exist (Oliver, 2010). Since then, VBNCs have been studied as a hidden risk of infection and antimicrobial resistance in various cases relevant to human health and wellbeing. The apprehension about the ability of both Gram-positive and Gram-negative bacteria to reach the VBNC stage and their associated risks began to permeate into the domain of the food industry and pharmaceutical sector since VBNCs were reported (Oliver, 2010). Bacteria may benefit from the VBNC state, but it is contrary for humans, since non-detection of live cells in the food sector, water supply system, or clinical settings might pose major concerns to the population (Du et al., 2007). Furthermore, VBNC cells may cause latency, and illness can return in treated individuals (Pai et al., 2000). Therefore, it is critical to know which human pathogens may reach the VBNC stage and to use reliable detection techniques to accurately measure the number of viable cells, incorporating both culturable, and VBNC cells.

In recent years, a deeper understanding of the live but non-culturable condition of bacteria has started evolving. Bacteria in this form are not detectable by conventional growth-based detection techniques. The transition to the VBNC stage has already been characterized and identified for many bacterial species majorly in relation to food-borne pathogens: *Escherichia coli* including (0157:H7), *Klebsiella* species, *Salmonella* species, *Helicobacter pylori, Campylobacter jejuni, Enterobacter* species, *Shigella* species, *Mycobacterium tuberculosis*, and many other species (Ramamurthy et al., 2014; Olaimat et al., 2019; Li et al., 2020b). *Norovirus, Salmonella, Clostridium perfringens, Campylobacter*, and *Staphylococcus aureus* are a few of the pathogens that have been identified by the CDC as being responsible for diseases that are caused by the consumption of food in the United States (Scallan et al., 2011). It is an established fact that *Salmonella, Campylobacter*, and enterohemorrhagic *E. coli* are bacteria that cause illness and even death in millions of people each year *via* food poisoning (Ali, 2011).

Infectious diseases caused by food-borne microorganisms are a major public health and food security problem. It not only poses risk to human health but also causes huge financial loss for the food processing industry. Till date, most of the existing studies have focused only on food-borne pathogens, especially those associated with the brewery industry. These well-designed studies elaborated on the effects of nutritional stress, acidity, temperature, and salt on food-borne pathogens to induce VBNC (Liu et al., 2017a,b,c, 2018a,2018b; Zhao et al., 2017; Schottroff et al., 2018; Li et al., 2020c,d; Xu et al., 2020, 2022; Busquets et al., 2022). Specifically, it has been shown in numerous studies that during food processing and preservation, *E. coli* can enter into the VBNC state (Oliver, 2010; Li et al., 2020c). Food stabilizing and sterilizing agents were already predicted to be a driver for transitioning into the VBNC state (Robben et al., 2018).

So, it is vital to examine the putative link between VBNC-state pathogens and food-borne infection outbreaks (Fakruddin et al., 2013), since methods intended to kill microorganisms might instead convert them to VBNC (Oliver, 2010). It has also been shown that it is possible to reverse the non-culturable stage to a growing stage with minor changes in the growth environment (Ramamurthy et al., 2014). A non-growing and merely viable state, VBNCs, have been repeatedly demonstrated to be able to revive or resuscitate thriving virulent bacteria once unfavorable triggers are removed (Li et al., 2014; Dong et al., 2020). Such survival instinct of bacteria has crucial implications in the food industry and healthcare settings. Precisely, concern in the scientific community started to accelerate because of the ability of VBNC to transform into an infectious form once inside a suitable host, and environment (Cappelier et al., 2005; Oliver, 2010). Subsequently, reports on food processing techniques, including pasteurizing milk, brewing beer, and chlorinating water, that facilitate the conversion of bacteria to the VBNC form further increased a vast interest in this topic (Oliver and Bockian, 1995; Liu et al., 2018b; Xu et al., 2020). The use of acetic acid as a food preservative along with incubation in a freezing temperature induced VBNC forms in *Pediococcus acidilactici* (Li et al., 2020a).

However, there is very little evidence regarding non-food related pathogens, which are clinically relevant and critical. However, in recent years, sporadic evidences are coming up to establish the role of ESKAPE pathogens in the food industry (Pérez-Rodríguez and Mercanoglu Taban, 2019; Li et al., 2020d; Khater et al., 2021). Recently, there are evidences of food being contaminated with ESKAPE pathogens in hospital settings (Patil et al., 2021). It has also been demonstrated that raw meat, fruit, and vegetables serve as a reservoir for *Acinetobacter baumannii, S. aureus*, and *Klebsiella pneumoniae* (Xu et al., 2012; Bao et al., 2017a,b; Pérez-Rodríguez and Mercanoglu Taban, 2019; Li et al., 2020d; Khater et al., 2021). The presence of virulence and MDR genes, as well as biofilm formation ability in these pathogens isolated from food, makes it more perilous to human health (Doughari et al., 2011; Carvalheira et al., 2017; Farouk et al., 2020; Cha et al., 2021; Elbehiry et al., 2021; Kumar et al., 2021). These isolates are not only detected in raw food, but also in baked foods even when stored under refrigeration with food preservatives (Peleg et al., 2009). The food isolates of *K. pneumoniae* and *A. baumannii* isolated from raw vegetables, street food, drinks, and raw and ready-to-eat (RTE) food from the supermarket were equally virulent as the clinical isolates from hospital patients (Hartantyo et al., 2020; Puspanadan et al., 2012; Budiarso et al., 2021). Since ESKAPE are known hospital pathogens, they could be a potential source of contamination in the hospital foods (Peleg et al., 2009; Malta et al., 2020), which were demonstrated in the hospital kitchen and visitor’s food (Campos et al., 2019), infant milk, and utensils in a public hospital (Araújo et al., 2015), and more recently, hospital water (Hayward et al., 2022).

Deducting from all this existing evidence, it is clear that if ESKAPE pathogens come in contact with a preservative-containing food in the hospital and get into a deep dormant state, it can pose a risk of evading detection and causing infection spread and the emergence of AMR. The possibility of VBNC induction poses a high risk of consumption of contaminated food even in a setting with stringent safety control processes, since the detection methods are based on growth culture.

Among FDA-approved food preservatives, formic acid (FA) is a major component that is responsible for antimicrobial effects *in vitro* and is widely used as a preservative in the food industry (Nabavi et al., 2015). Though many studies exist as a preview of VBNC induction in food-borne pathogens (Schottroff et al., 2018), direct evidence regarding the ability of food stabilizing agents in inducing VBNC of ESKAPE pathogens and subsequently resuscitating them into an altered virulent and resistant state is the need of the hour.

Therefore, in the present study, we have explored for the first time, the ability of the widely used food preservative FA to induce VBNC in various physical conditions for human pathogens mostly abundant in hospital settings. Through this study, we have demonstrated induction of VBNC, validation of VBNC, and also showed its resuscitation after removal of FA in two model species *A. baumanii* and *K. pneumoniae*. After resuscitation, AMR phenotype and virulence gene expression increased significantly when compared to the original isolate, validating the critical impact of this study.

## Materials and methods

### Microbial strains and growth conditions

In the present study, clinical isolates of *A. baumannii* (AB-16) and *K. pneumoniae* (BA19058) have been used for VBNC induction and characterization. Both isolates were retrieved from the blood of sepsis patients. Since this is the first report of VBNC induction in *A. baumannii* and *K. pneumoniae*, we have also used a non-pathogenic isolate of *E. coli* (ATCC-Dh5α) as a model organism to validate the process and condition of VBNC induction. *A. baumannii* and *E. coli* have been cultured in the 2XYT broth (HiMedia, Mumbai, India) while *K. pneumoniae* was cultured in MHB media (HiMedia, Mumbai, India). A master cell bank of each isolate was stored at -80^°^C. Before each experiment, a fresh aliquot of glycerol stock was revived in respective media and followed by the subculture at 37^°^C with shaking at 250 rpm.

### Formic acid treatment

To optimize VBNC induction, 10^9^ cells/ml from the mid-log phase of secondary culture was centrifuged at 4,000 rpm for 10 min. According to FDA, the maximum approved FA concentration is 2.1% (Gras Notice Inventory [FDA], 2022). The concentration of FA was optimized for 1–3%. In the case of *A. baumannii* and *K. pneumoniae*, a few growing colonies were observed at 1 and 2% concentrations of FA at 4^°^C. However, 3% FA induced VBNC at all three storage temperatures in all three bacterial species (*A. baumannii, K. pneumoniae*, and *E. coli*). Therefore, in order to maintain uniformity in further characterization studies, 3% FA was taken forward for all other experiments.

Pellet obtained from the growing culture was resuspended in 3% FA-containing media and incubated at 4, 25, and 37^°^C without shaking. To compare the effect of FA on VBNC induction, cells in media without FA were used as growing control (without FA) and cells killed (without media) were used as non-viable control. A 200 μl aliquot from each treatment was then tested for VBNC induction on day 2, day 4, and day 10 in all three species. On day 10 after FA removal, in-depth VBNC characterization was done by validation through membrane integrity assessment and *de novo* ATP estimation.

### Assessment of viable-but-non-culturable induction

To investigate VBNC induction, four different properties of the VBNC state of bacteria were used (Colwell, 2009). As previously described (Colwell, 2009), during the VBNC state, bacteria lose their culturability. So, culturability was checked to assign VBNC status on day 2 and day 4. In-depth VBNC characterization on day 10 was done by testing culturability and metabolic viability (ATP levels) at 0, 2, 4, and 24 h after FA removal. Additionally, cell viability was detected by using respiratory activity marker 5-cyano-2,3-ditolyl-tetrazolium chloride (CTC) flow cytometry and propidium monoazide (PMA) PCR. Earlier, VBNC bacteria were detected either on the basis of culturability and energy production or membrane permeability and respiration. In the present study, we have utilized all four parameters for the validation of the VBNC state.

### Bacterial growth detection

The culturability of bacteria was checked by the standard agar plate method. The culture was spread on agar plates on days 2, 4, and 10 immediately after the removal of FA. While on day 10, further culturability was tested up to 4 h of incubation in fresh media at 37^°^C with shaking at 250 rpm. The plates were incubated at 37^°^C for 18–24 h, and the image was taken using Chemidoc (Bio-Rad, Hercules, CA, United States). No colony formic unit (CFU) culture was considered for further experiments for viability.

### Metabolic activity during viable-but-non-culturable

To assess the metabolic activity of bacteria in the VBNC state, ATP production was quantified by using a commercially available assay (BacTiter-GloTM Microbial Cell Viability Assay, Promega, Madison, WI, United States) as per the manufacturer’s protocol. In short, an equal volume of BacTiter mixture was mixed with an equal volume of bacterial culture followed by 5 min of incubation at 37^°^C. The relative light unit (RLU) was measured by using BioTek Synergy HTX Multimode Reader (Agilent, Santa Clara, CA, USA). The luminescence value for media only (negative control - without cells) wells was used as a background luminescence and was subtracted to get the actual RLU reading.

### Fluorescence-based viability determination

The qualitalive analysis of the respiratory marker of viability was done by using BacLight™ RedoxSensor™ CTC Vitality Kit (Invitrogen, Waltham, MA, United States) on a confocal microscope, and the same assay was performed for quantitative analysis in a flow cytometer based on the manufacturer’s protocol. Briefly, the assay is based on the principle that viable cells must respire *via* an electron transport system, and the transport of electrons can reduce the CTC into red fluorescent formazan. Counter stain with DAPI can differentiate dead cells from cell debris. For comparison, 1 ml of 10^6^ cells from actively growing culture (positive control, growing control without any FA) and culture killed with a fixative (4% formaldehyde) solution (negative control and non-viable control) were used. For staining, cells were first centrifuged and washed two times with sterile 1X PBS. Then it was resuspended in 10 mM glucose containing 1x PBS, containing 5 mM CTC (final concentration), and incubated at 37^°^C for 30 min. CTC staining was followed by fixing with 4% formaldehyde for 30 min at 37^°^C and counter staining with 10 μg/ml DAPI (4’,6-diamidino-2-phenylindole, dihydrochloride). For quantitation, stained cells were then passed through flow cytometry Canto II (BD bioscience Franklin Lakes, NJ, USA). For CTC detection 488/585 nm and for DAPI at 405/440 nm, excitation/emission filters were used. The unstained culture was used to set the instrument baseline, and comparative quantitation was done by using growing and non-viable control. For qualitative microscopic comparison, 2 μl of stained culture was smeared on a glass slide, sealed, and analyzed under confocal laser scanning microscopy (CLSM). Since growing cells are more metabolically active than the VBNC cells, images were captured at different settings for growing control vs. VBNC to prevent signal saturation and ensure optimal detection.

### Membrane integrity determination

Changes in cell membrane integrity as a result of FA treatment were investigated by using PMA (Biotium, San Francisco, CA, USA), according to the manufacturer’s protocol. Briefly, each sample was divided into two groups: one was treated with PMA while another was not. For PMA treatment, 10^7^ cells were mixed with 25 μM of PMA and incubated at 37^°^C for 20 min in the dark. For ensuring covalent binding of PMA with DNA, samples were exposed to halogen light (600 W blub) for 20 min in a temperature-controlled chamber to prevent cell damage because of heating. After removing PMA by spinning the cells at 4,000 rpm for 20 min, DNA was extracted by conventional phenol-chloroform-isoamyl alcohol method and stored at -20^°^C till further use. Two sets of previously reported universal 16S rRNA primers (forward-1 - 5’-ACTCCTACGGGAGGCAGCAG-3’, reverse-1 - 5’-GGCGTGGACTACCAGGGTATC-3’; forward-2 - 5’-CAGGATTAGATACCCTGGTAGTCC-3’ and reverse-2 5’-TGACGGGCGGTGTGTACAAG-3’) were used for real-time PCR (Barghouthi, 2011). Quantitative data analysis was done by the delta-delta Ct-value method, as per the manufacturer’s instructions.

### Resuscitation from viable-but-non-culturable state

In the present study, we have also demonstrated resuscitation from the VBNC state by adding fresh media, spent media, and fresh media with a combination of spent media (1:1). A freshly prepared cell-free supernatant of the mid-log phage of actively growing culture from the same isolate is being referred to as “spent media” in this study. The supernatant was harvested from culture by centrifugation at 4,000 rpm for 20 min. Then cell-free supernatant was filtered for two times with 0.45 and 0.1 μm sterile disposable syringe filters. For resuscitation, FA was first removed from the cells, and cells were washed three times with sterile phosphate buffer. Then the cells were re-suspended at 10 times the volume of fresh media (M), spent media (S), and fresh media plus spent media (SM). The culture was then incubated at 37^°^C and 250 rpm shaking for up to 48 h. The culturability of the bacteria was tested by spotting 10 μl of culture on an agar plate at 0, 2, 4, 18, 24, and 48 h. Spotted plates were incubated at 37^°^C for 18–24 h, and the image was captured by Chemidoc for CFU count.

### Analysis of antimicrobial susceptibility profile before and after viable-but-non-culturable induction

Furthermore, the antimicrobial susceptibility (AST) pattern was tested before and after FA treatment. Because AST is a growth-based method, we performed it only on actively growing and resuscitated bacteria. VBNC state could not be utilized for phenotypic AST profiling by disk diffusion method because of its non-culturability. For AST profiling, the standard disk diffusion method was used, following CLSI guidelines. Thirty-one different antibiotic disks with manufacturer-specified concentrations (Himedia, Mumbai, India) were used for this experiment (Supplementary Table 2). About 100 μl of 10^4^ cells/ml (following CLSI alternate optical method of using 0.08–0.13 OD turbid suspension at 625 nm) was spread on a freshly prepared agar plate, and antibiotic disks were placed. The plate was then incubated at 37^°^C overnight, and the diameter of the zone of inhibition was measured for further comparative analysis.

### Gene expression analysis of AMR and virulence genes

For the detection of AMR and virulence gene expression, primers were designed for multiple AMR and virulence genes based on standard annotation. Suspensions of 10^8^ cell/ml bacteria of growing, VBNC, and resuscitated state were lysed in 1 mg/mL lysozyme containing Tris-EDTA buffer (pH 8.0). After incubation at 37^°^C for 10 min, total RNA was extracted from cells using a column-based RNA extraction Kit (Macherey-Nagel, Düren, Germany). RNA was quantified by using a spectrophotometer (Thermo, Waltham, MA, United States). cDNA was synthesized using the iScript cDNA Synthesis kit (Bio-Rad, Mumbai, India), and gene expression was quantified by real-time PCR (qRT-PCR) using iTaq UniverSYBR Green SMX (Bio-Rad, Mumbai, India). Different genes for efflux pump, porins, and virulence factors were selected for quantitative analysis by the delta Ct-value method by normalizing the expression with 16S rRNA expression levels.

### Statistical analysis

All the experiments were performed independently three times with three replicates in each, and results are shown with standard error of means (SEM) as error bars. Statistical significance (significance level of *p* ≤ 0.05) was calculated by GraphPad Prism software (version 8.0, GraphPad Software, San Diego, CA, USA) using two-way ANOVA and Tukey *post-hoc* test.

## Results

### Viable-but-non-culturable detection

**FIGURE 1:**
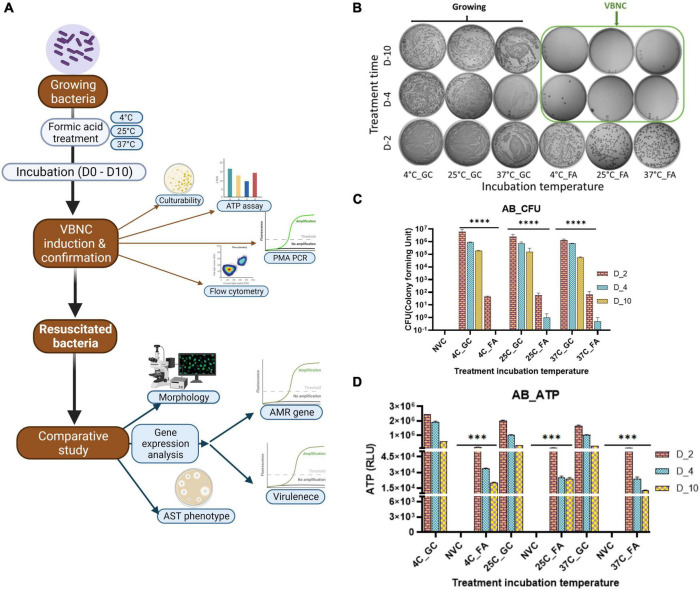
Viable-but-non-culturable (VBNC) optimization VBNC induction after formic acid (FA) treatment was checked on day 2, day 4, and day 10. VBNC induction was confirmed by using two properties of the VBNC state, non-culturability and metabolic energy production, in *Acinetobacter baumannii* (*Klebsiella pneumoniae* and *E. coli* refer Supplementary Figure 1) with FA treatment at three different incubation temperatures, that is, 4, 25, and 37^°^C. The study work flow representation **(A)** VBNC induction optimization in *A. baumannii* culturability assessment, **(B)** quantitative representation of culturability **(C)**, and ATP production **(D)**. All three study isolates show growth on day 2 but the loss of culturability on day 4–day 10 **(B)**. This reduction of growth in VBNC was highly significant when compared to untreated growing bacteria from the same incubation temperature while non-significant in non-viable bacteria **(C)**. Non-growing bacteria on day 4–day 10 showed significant ATP production when compared to non-viable bacteria in all three study isolates **(D)**. FA, formic acid; GC, growing control; NVC, non-viable control; 4C, sample incubated at 4^°^C; 25C, sample incubated at 25^°^C; 37C, sample incubated at 37^°^C; D_2, sample tested after 2 days of FA treatment; D_4, sample tested after 4 days of FA treatment; D_10, sample tested after 10 days of FA treatment; CFU, colony formic unit; RLU, relative light unit; AB, *Acinetobacter baumannii*, ATP- Adenosine triphosphate, **P-valve < 0.05, and ***P-valve < 0.005.

**FIGURE 2:**
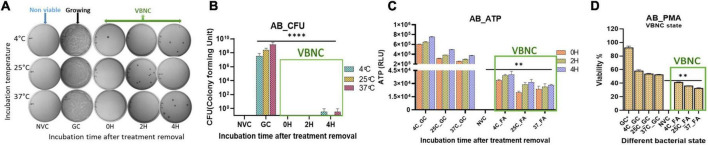
Viable-but-non-culturable (VBNC) validation after VBNC induction was further confirmed on day 10. VBNC induction by culturability and ATP production up to 4 h after formic acid (FA) removal and addition of fresh media. Furthermore, viability percentage was checked on the basis of membrane integrity in all the study isolates and at all three incubation temperatures. VBNC induction confirmation in *Acinetobacter baumannii* (*Klebsiella pneumoniae* and *E. coli* refer Supplementary Figure 2) culturability detection **(A)**, quantitative representation of culturability **(B)**, ATP production **(C)**, and propidium monoazide (PMA) PCR assay **(D)**. FA-induced VBNC at all three incubation temperatures did not resume detectable growth up to 4 h after removal of FA and incubation under optimal growth conditions. After removal of FA, higher ATP levels indicated *de novo* metabolic energy production in non-culturable state of *A. baumannii* **(C)**. PMA PCR results showed up to 45% population maintained intact cell membranes in the VBNC state **(D)**. FA treatment in combination with 4^°^C incubation induced more VBNC forms when compared to 25 and 37^°^C. While viability percentage of 37^°^C incubation was significant when compared to the non-viable control. FA, formic acid; GC, growing control; NVC, non-viable control; 4C, sample incubated at 4^°^C; 25C, sample incubated at 25^°^C; 37C, sample incubated at 37^°^C; D_2, sample tested after 2 days of FA treatment; D_4, sample tested after 4 days of FA treatment; D_10, sample tested after 10 days of FA treatment; CFU, colony formic unit; RLU, relative light unit; AB, *Acinetobacter baumannii*, **P-valve < 0.05, and ****P-valve < 0.0005.

To maintain viability, bacteria have to maintain the basal ATP level. The ATP level was tested on days 2, 4, and 10 for *A. baumannii*, *K. pneumoniae*, and *E. coli*. Treatment with FA for 10 days is sufficient for maintaining optimum viability. So, on day 10, ATP production was tested after the removal of FA, followed by fresh media addition and incubation at 37°C with shaking at 250 rpm for up to 4 h. ATP level decreased on day 4 through day 10, but it remained significantly higher than the non-viable control; however, on day 2, bacteria showed a higher number of culturable cells, but culturability drastically declined from day 4 to day 10. ATP production decreased continuously. That may indicate that longer treatment time induces a deeper dormant state with decreased metabolic rate. On the 10th day, ATP levels increased after 2 and 4 h of incubation at optimal growth conditions after the removal of FA. The culturability and ATP production are shown in *A. baumannii* (Figures 1, 2), *K. pneumoniae*, and *E. coli* (Supplementary Figures 1, 2).

### Viability estimation by determining respiration intensity and membrane integrity

**FIGURE 3:**
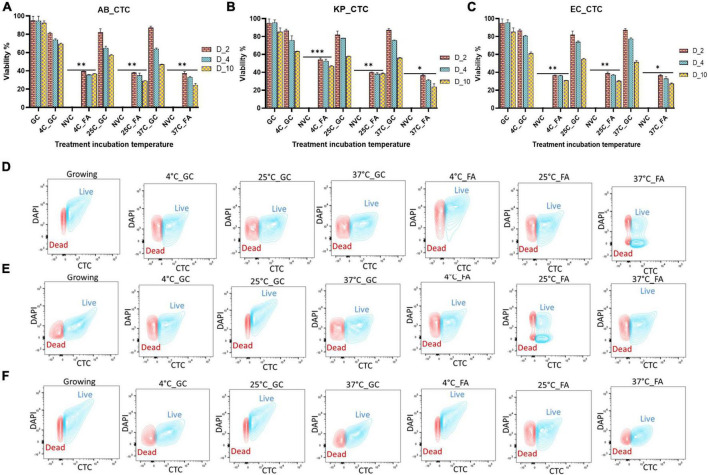
Determination of respiratory activity as a viability marker after formic acid (FA) treatment. After viable-but-non-culturable (VBNC) confirmation, the respiratory activity of bacteria at the VBNC state was further measured by the flow cytometry on day 2, day 4, and day 10 in *Acinetobacter baumannii* **(A)**, *Klebsiella pneumoniae* **(B)**, and *E. coli* **(C)** at incubation temperatures of 4, 25, and 37^°^C. Flow cytometry of untreated and treated cells on day 10 is also shown here in three study isolates *Acinetobacter baumannii* **(D)**, *Klebsiella pneumoniae* **(E)**, and *E. coli* **(F)**. (For day 2 and day 4 data, refer to Supplementary Figure 3). A similar proportion of cells are viable on day 4 and day 10 as compared to day 2, but on day 2, cells are growing but they lost viability after day 4. Among all three temperatures, the viability percentage decreased at 4, 25, and 37^°^C gradually. Though VBNC induction starts on day 4, there is no significant difference in the viability of bacterial population on day 2 vs. days 4–10. VBNC viability is significant to non-viable control. FA, formic acid; GC, growing control; NVC, non-viable control; 4C, sample incubated at 4^°^C; 25C, sample incubated at 25^°^C; 37C, sample incubated at 37^°^C; D_2, sample tested after 2 days of FA treatment; D_4, sample tested after 4 days of FA treatment; D_10, sample tested after 10 days of FA treatment; AB, *Acinetobacter baumannii*; KP, *Klebsiella pneumoniae*; EC, *E. coli*; growing, actively growing culture from mid-log phage; 4C_GC, 4C culture without treatment at 4^°^C; 25C_GC, 25^°^C culture without treatment at 25^°^C; 37C_GC, 37^°^C culture without treatment at 37^°^C; 4C_FA, 4^°^C culture with treatment at 4^°^C; 25C_FA, 25^°^C culture with treatment at 25^°^C; 37C_FA, 37^°^C culture with treatment at 37^°^C. **P-valve < 0.05, ***P-valve < 0.005, and ****P-valve < 0.0005.

**FIGURE 4:**
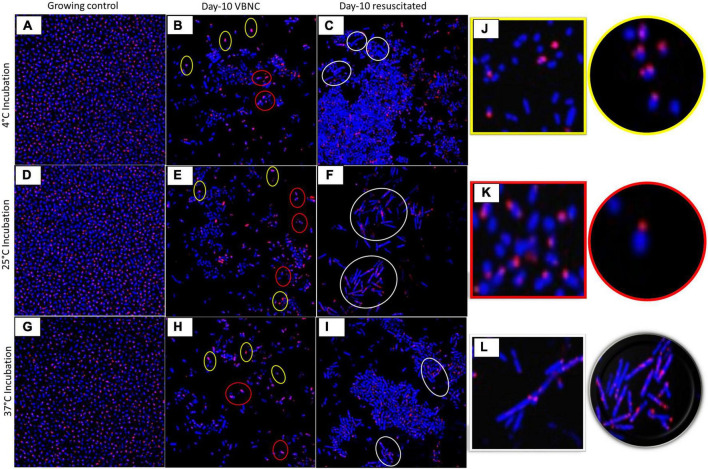
Morphological changes of viable-but-non-culturable (VBNC) and resuscitation state. Morphology of VBNC and resuscitated state in terms of shape and size was compared with growing bacteria under confocal laser scanning microscopy (CLSM) in *Acinetobacter baumannii* in different growth conditions at 4^°^C **(A–C)**, 25^°^C **(D–F)**, and at 37^°^C **(G–I)** (for *Klebsiella pneumoniae* and *E. coli*, refer Supplementary Figures 4–6). Growing control bacteria **(A,D,G)**, Day 10 VBNC **(B,E,H)**, and Day 10 resuscitated **(C,F,I)** at temperature 4,25 and 37^°^C. FA treated day 4 VBNC and resuscitated (refer Supplementary Figure 4). Morphology altered in VBNC and resuscitated state **(J)** Yellow highlight shows cocci-shaped bacteria, **(J)** red highlight shows reduced bacilli, **(K)** white highlight shows elongated cells **(L)**. All cells stained with DAPI showed blue color, and CTC-tagged live cells give red color. Bacteria in the VBNC state are smaller in size and become shorter rods and cocci as compared to the growing state (**J**, yellow and **K**, red highlight). After resuscitation, bacteria became shrunken and elongated when compared to growing bacteria (**L**, white highlight).

Measurement of bacterial respiration is considered to be one of the important criteria to detect viable cells (Morishige et al., 2015). The respiratory activity of FA-treated *A. baumannii*, *K. pneumoniae*, and *E. coli* was estimated by the standard CTC reduction assay (Cappelier et al., 1997). The detection of CTC was performed by both flow cytometry and confocal microscopy. The viability of the untreated group declined from day 2 to day 10 from ∼90 to ∼50%, while the viability percentage of the treated group was ∼50% on day 2 and remained almost constant till day 10, independent of incubation temperature. The pattern of viability reduction was similar in all three bacterial species with minor variations (Figure 3 and Supplementary Figure 3). Cell viability was also determined on the basis of membrane integrity by using PMA dye. A combination of membrane impermeability and strong DNA-binding ability enables PMA to be used as a viability marker (Nocker and Camper, 2006; Ge et al., 2019). In the last few years, qPCR is being increasingly used for the detection of viability and membrane integrity in combination with DNA-binding dye PMA (Zhong et al., 2016, 2018; Bao et al., 2017b; Liu et al., 2018b; Zhao et al., 2020). Membrane integrity-based viability showed a similar pattern as shown by respiratory-based marker assay (Figure 2 and Supplementary Figure 2). Cell viability and morphological changes were then visualized under a confocal microscope at 60X oil immersion magnification. All cells (dead and live) were stained with DAPI (blue), while CTC stain (red) tagged only viable cells. A comparative study of growing and VBNC bacteria showed morphological discrepancies in *A. baumannii*, *K. pneumoniae*, and *E. coli*. When compared to actively growing homogenous culture, VBNC culture was a heterogenous population, and the majority of the cells were smaller in size and transformed from bacilli to coccoid. Furthermore, the resuscitated culture showed increased homogeneity with more elongated cells when compared to VBNC and growing state. The cells of *E. coli* in their VBNC form were noticeably shorter when compared to the VBNC state of *A. baumannii* and *K. pneumoniae*. This demonstrates that there are strain-dependent changes in the shape and morphology of the cells attaining the VBNC state (Figure 4 and Supplementary Figures 4–6).

### Resuscitation from viable-but-non-culturable state

**FIGURE 5:**
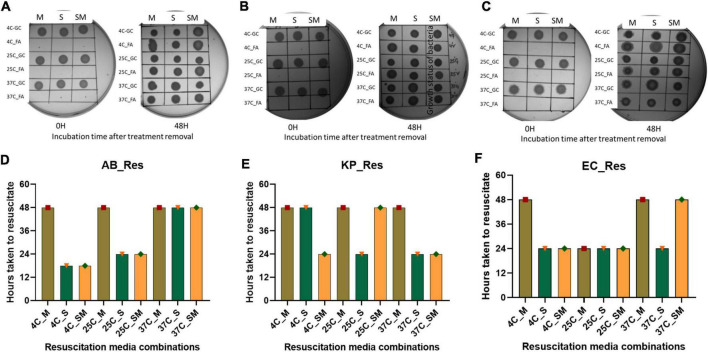
Resuscitation of bacteria from viable-but-non-culturable (VBNC) state. VBNC bacteria was resuscitated by removal of formic acid (FA) treatment and addition of fresh media, spent media, and a combination of fresh and spent media (1:1) in this figure represents the growth of bacteria on agar plate and time taken for resuscitation. Cultural growth representation of Acinetobacter baumannii **(A)**, Klebsiella pneumoniae **(B)**, and *E. coli* **(C)** at 37^°^C incubation. Quantitative representation of Resuscitation was determined on the basis of time taken after removal of FA, Acinetobacter baumannii **(D)**, Klebsiella pneumoniae **(E)**, and *E. coli* **(F)** at 37^°^C incubation. Addition of spent media reduced the resuscitation time to 18 h which was formerly 48 h. FA, formic acid; GC, growing control; NVC, non-viable control; 4C, sample incubated at 4^°^C; 25C, sample incubated at 25^°^C; 37C, sample incubated at 37^°^C; D_2, sample tested after 2 days FA treatment; D_4, sample tested after 4 days of FA treatment; D_10, sample tested after 10 days of FA treatment; AB, *Acinetobacter baumannii*; KP, *Klebsiella pneumoniae*; EC, *E. coli*; M, fresh media; S, spent media; SM, fresh media + spent media.

Revival of growth of VBNC bacteria after removal of environmental stress is important to validate the true VBNC state. For resuscitation, several strategies have been followed by researchers, and the most common one is the removal of stress conditions (Gupte and Joseph, 2001). While in some studies, additional strategies were adopted to induce the resuscitation, e.g., dilution of cells (Whitesides and Oliver, 1997; Zhang et al., 2016), addition of EDTA (Kan et al., 2019), spent media (FernÁndez-Delgado et al., 2015), etc. For resuscitation, we used a stress removal strategy and supplantation with spent media. Removal of stress and addition of fresh media resumed growth within 48 h. The spent media and spent media in combination with fresh media reduced resuscitation time by 18 h. Overall, VBNCs induced by specific food preservatives at different storage temperatures resumed growth within 48 h of FA removal (Figure 5).

### Antimicrobial susceptibility pattern

**FIGURE 6:**
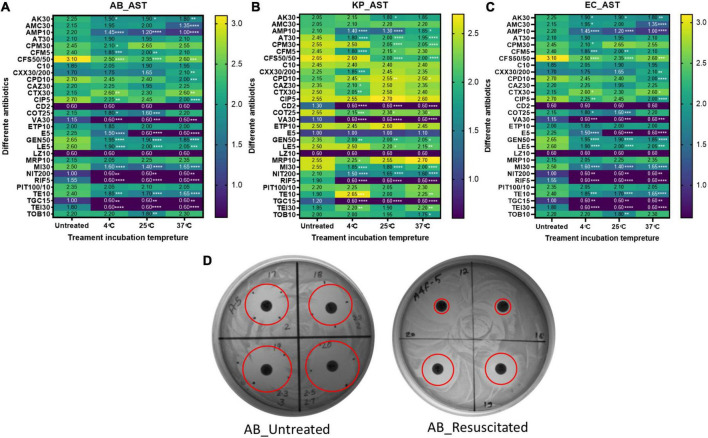
AST profiling. The antimicrobial susceptibility pattern of resuscitated bacteria was checked by the disk diffusion method. AST pattern on the basis of the zone of inhibition diameter among untreated and formic acid (FA)-treated cells at 4, 25, and 37^°^C for panel **(A)** *Acinetobacter baumannii*, **(B)** *Klebsiella pneumoniae*, and **(C)** *E. coli* are shown here. Panel **(D)** is a representation of the disk diffusion agar plate of FA treatment at 4^°^C in *A. baumannii* with reduced clear zone diameter (in cm) in resuscitated bacteria. Statistical significance of diameter changes was determined by two-way ANOVA with multiple comparison followed by correction with Dunnett’s multiple comparison test using GraphPad Prism version 9.0.0. FA, formic acid; GC, growing control; NVC, non-viable control; 4C, sample incubated at 4^°^C; 25C, sample incubated at 25^°^C; 37C, sample incubated at 37^°^C; AB, *Acinetobacter baumannii*; KP, *K. pneumoniae*; EC, *E. coli*; AK30, Amikacin (disk); AMC30, Amoxiclav (Amoxicillin/Clavulanic acid) disk; AMP-10, Ampicillin (disk); AT-30, Aztreonam disks; CPM50, cefepime disk; CFM5, Cefixime (disk); CFS 50/50, Cefoperazone (Sulbactam) disks; C10, Cefotaxime disks; CXX 30/200, Cefoxitin/Cloxacillin disks; CPD10, Cefpodoxime disks; CAZ30, Ceftazidime (disk); CTX30, Chloramphenicol disks; CIP5, Ciprofloxacin (disk); CD2, Clindamycin (disk); CO25, Co-Trimoxazole (sulfa/trimethoprim) disks; VA30, Vancomycin (disk); ETP10, Ertapenem (disk); E5, Erythromycin-1V disk; GEN50, Gentamycin (disk); LE5, Levofloxacin disk; LZ10, Linezolid (disk); MRP10, Meropenem (disk); MI30, Minocycline disk; NIT200, Nitrofurantoin disks; RIF-5, Rifampicin disks; PIT 100/10, Tazobactam/Piperacillin disks; TE 10, Teicoplanin disks; TGC-15, Tigecycline (disk); TOB 10, Tobramycin disk; TEI 30, Tetracycline (disk). *Represents *P*-valve < 0.5, **P-valve <0.05, ***P-valve < 0.005, and ****P-valve < 0.0005.

Although a few, there are reports on prolonged persistence and different xenobiotic resistance pattern of bacteria in VBNC, as well as in a resuscitated state, when compared to growing bacteria. We checked the phenotypic AST pattern of resuscitated bacteria in comparison with the untreated growing culture. All three species show an extended resistance pattern after resuscitation compared to the untreated growing control (Figure 6). In the case of *E. coli*, the AST profile of these antibiotics (AK30, AMP-10, CFS50/50, VA30, E5, GEN50, LE5, MI10, NIT200, RIF-5, TE10, TGC-15, and TEI30) shows significant changes in all treatment conditions. In the case of *A. baumannii*, the AST profiles of AK30, AMP-10, CFS50/50, VA30, E5, GEN50, LE5, MI30, NIT200, RIF-5, TE10, TGC-15, and TEI30 changed. In the case of *K*. *pneumoniae*, AMP-10, AT-30, CTX30, CD2, COT25, VA30, GEN50, MI30, NIT200, RIF-5, TGC-15, and CFS50/50 were changed. These are the list of antibiotics for which AST patterns changed significantly in all the treatment temperatures. But for the other few antibiotics, sporadic changes were found, which is mentioned in Figure 6. The clear zone diameter of AMP-10, CFS50/50, E5, GEN50, LE5, MI30, NIT200, RIF-5, TGC-15, and TEI30 was significantly reduced in all the isolates. Other antibiotics like C10, CAZ10, ETP10, MRP10, and PIT100/10 showed no change in the zone of clearance after treatment in all three bacteria.

### Change in expression of genes majorly responsible for new AMR traits

**FIGURE 7:**
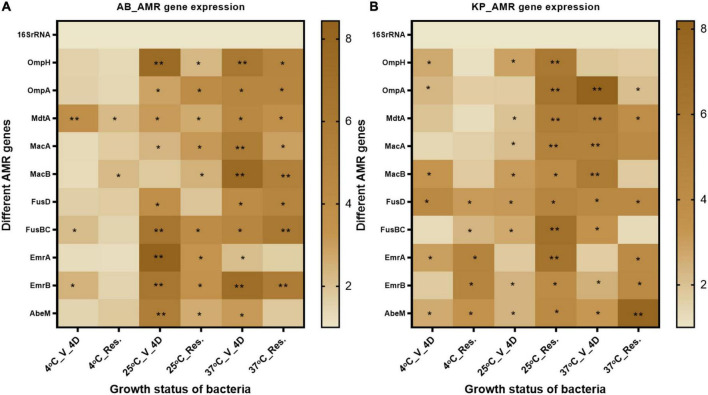
AMR gene expression- Efflux pump and outer membrane proteins related to gene expression were checked after 4-day formic acid (FA) induced VBNC and resuscitated bacteria. **(A)** The expression pattern in *Acinetobacter baumannii*, and **(B)** expression pattern in *Klebsiella pneumoniae*. Efflux pump genes related to the antibiotics in VBNC as well as in resuscitated cells at 25 and 37^°^C (4 days) were increased up to 10-fold. While almost 2–4-fold expression was observed in VBNC and resuscitated cells incubated at 4^°^C. The statistical significance of fold change was determined by two-way ANOVA with multiple comparison followed by correction with Dunnett’s multiple comparison test using GraphPad Prism version 9.0.0. V, VBNC; Res, resuscitated; 4C_V_4D, day 4 VBNC at 4^°^C and FA; 25C_V_4D, day 4 VBNC at 25^°^C and FA; 37C_V_4D, day 4 VBNC at 37^°^C and FA; 4C_Res_4D, day 4 resuscitated from 4^°^C and FA induced VBNC; 25C_Res_4D, day 4 resuscitated from 25^°^C and FA induced VBNC; 37C_Res_4D, day 4 resuscitated from 37^°^C and FA induced VBNC; 4C_V_10D, day 10 VBNC at 4^°^C and FA; 25C_V_10D, day 10 VBNC at 25^°^C and FA; 37C_V_10D, day 10 VBNC at 37^°^C and FA. *P-valve < 0.5, **P-valve < 0.05.

It was not feasible to assess phenotypic AST patterns in the VBNC state due to the limitation of non-culturability. Based on the existing knowledge of the mode of action of FA, it was hypothesized that the resistance pattern might be altered due to the modified expression of efflux pumps. So, in the present study, we investigated the expression of efflux pumps and porins to see if these reflect the AMR phenotype change. We investigated the expression of outer membrane porins OmpH and OmpA, ABC efflux pumps MacAB and FusBCD, and major facilitator superfamily (MSF) efflux pump EmrAB (Supplementary Table 1). The expression of efflux pumps and porins was significantly elevated after FA treatment, and was maintained at high levels even after resuscitation (Figure 7). In *A. baumannii*, the expression of outer membrane proteins, MSF, and ABC family efflux pump, was highly upregulated in VBNC and resuscitated state at 25 and 37^°^C when compared to 4^°^C incubation. In the case of *K. pneumoniae*, the expression of outer membrane protein and ABC family efflux pump was more enhanced at 37^°^C and resuscitated state at 25 and 37^°^C. In *K. pneumoniae*, MSF genes showed significantly increased expression after resuscitation.

### Change in virulence gene expression

**FIGURE 8:**
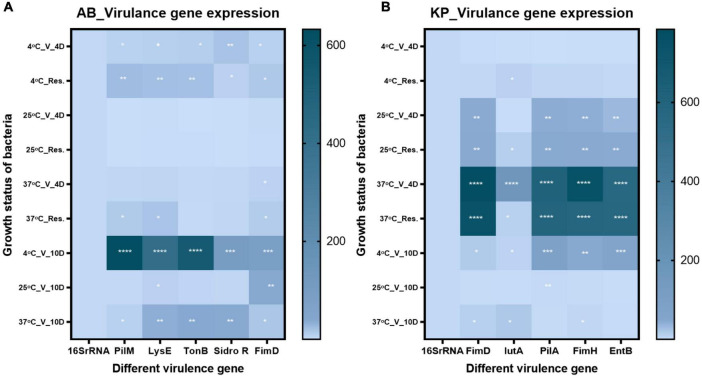
Virulence gene expression Virulence gene expression was checked in viable-but-non-culturable (VBNC) state after 4- and 10 day treatment and resuscitated bacteria from 4 day VBNC. **(A)** The expression pattern in *Acinetobacter baumannii* and **(B)** expression pattern in *Klebsiella pneumoniae*. Upregulation of virulence genes in VBNC and resuscitated forms incubated at 4–37^°^C on days 4 and 10 was observed. While constant expression of virulence genes was observed in 25^°^C incubated VBNC and resuscitated forms. The statistical significance of fold change was determined by two-way ANOVA with multiple comparison followed by correction with Dunnett’s multiple comparison test using GraphPad Prism version 9.0.0. V, VBNC; Res, resuscitated; 4C_V_4D, day 4 VBNC at 4^°^C and FA; 25C_V_4D, day 4 VBNC at 25^°^C and FA; 37C_V_4D, day 4 VBNC at 37^°^C and FA; 4C_Res_4D, day 4 resuscitated from 4^°^C and FA induced VBNC; 25C_Res_4D, day 4 resuscitated from 25^°^C and FA induced VBNC; 37C_Res_4D, day 4 resuscitated from 37^°^C and FA induced VBNC; 4C_V_10D, day 10 VBNC at 4^°^C and FA; 25C_V_10D, day 10 VBNC at 25^°^C and FA; 37C_V_10D, day 10 VBNC at 37^°^C and FA. *P-valve < 0.5, **P-valve < 0.05, ***P-valve < 0.005, and ****P-valve < 0.0005.

We examined the expression of five different virulence genes in *K. pneumoniae* and *A. baumannii* strains. The genes estimated for *A. baumannii* were tonB, sideroF, fimD, pilM, and lysE. The genes for *K. pneumoniae* were IutA, fimH, fimD, entB, and pilA (Supplementary Table 1). The gene expression of VBNC state on day 4 and day 10 was examined along with resuscitated population on day 4. In *A. baumannii*, VBNC state and resuscitated state at 4^°^C on day 4 and day 10 showed a significant upregulation in the above-mentioned virulence genes. At 37°C, VBNC state on day 4 and day 10, whereas 25^°^C VBNC state on day 4 and day 10 and resuscitated state on day 4 showed constant expression. In the case of *K. pneumoniae*, temperatures of 4, 25, and 37^°^C degrees resuscitated, and VBNC condition on day 4 exhibited upregulation of virulent genes. On day 10, the VBNC state exhibited upregulation only at 4 and 37^°^C, whereas constant expression was observed at 25^°^C showed (Figure 8). Overall, in most of the conditions, virulence genes were upregulated.

## Discussion

Food-borne pathogens in VBNC are a hazard to public health and food safety, as they cannot be identified by culture-based food and water testing methods. It was discussed earlier that the VBNC condition may explain why 80% of food-borne infections have no identified cause (Li et al., 2014). Research on the VBNC state in microbes is critically needed to address food safety concerns, as well as the need to understand the processes of VBNC induction, its resuscitation, and regulation of virulence and AMR in the process.

Food processing transforms and stabilizes food and improves shelf life, for more efficient transport and distribution, and is an essential component of the current lifestyle all over the world. The processing involves physical methods like temperature and pressure and the use of various chemical agents. Temperature monitoring is critical to prevent bacterial proliferation. Keeping the food below recommended temperatures, wherein bacteria can grow, may substantially reduce food spoiling, and food-borne infections. Lower temperatures slow down the natural processes occurring inside the cells, driving the cells to deep dormancy in many bacteria (Wei and Zhao, 2018). Food storage methods also use salt, sugar, and organic acids. Weak organic acids (acetic, formic, lactic, sorbic, benzoic, propionic, maleic, tartaric, citric, and ascorbic) affect the pH of bacterial cells. FA is an antimicrobial preservative, permitting fermentation at lower temperatures and avoiding nutrient loss (Peh et al., 2020). In the present study, VBNC was induced by using FA, which supports previous evidence that many human pathogens enter the VBNC state when they are exposed to environmental stressors that are often seen in clinical settings or food processing facilities (Amchova et al., 2015). Studies reported that at low doses, feed additives/preservatives, and sanitizers transform *E. coli* into a VBNC condition (Ogane et al., 2019). In the present study, FA induced VBNC after 4-day treatment, while these populations remained viable for up to 10 days, which is similar to a previous finding in *E. coli* cells in seawater, where the cells were viable for up to 30 days (Ogane et al., 2019). In our study, the viable population remained significant at all three incubation temperatures (4, 25, and 37^°^C), as was earlier shown for citric acid at 4^°^C in *E. coli* (Wei and Zhao, 2018).

Organic acids are popular food additives as pathogen blockers. These organic acids are used as food supplements to reduce infections and improve Gut functioning. Feed additions including organic acids, such as acetate, propionate, and butyrate, have received a lot of attention; however, FA, though widely used, is yet to obtain due consideration for safety. On the basis of investigations conducted using *in vitro* models, it has been shown that FA is a reasonably efficient antibacterial agent against *Salmonella spp*. and other infections (Michiels et al., 2012).

Formic acid is authorized by FDA and also listed in the European Union register of food additives for use in the organic production of agricultural food and as a food packaging additive. The Joint FAO/WHO Expert Committee on Food Additives established an acceptable daily intake (ADI) of FA of 0–3 mg/kg BW (Gras Notice Inventory [FDA], 2022). Once within a bacterial cell, in which the pH is 7, FA dissociates and lowers the cytoplasmic pH, inactivating decarboxylases and catalases (Luise et al., 2020). It is dependent on the pKa of the acid to infiltrate the bacterial cells, since the acid may enter the surrounding bacterium in its undissociated state.

Numerous previous studies have concluded that FA is the major component that is responsible for antimicrobial effects *in vitro* in the food industry (Nabavi et al., 2015). Though on the other hand, different bacterial strains, such as *E. coli, S. Typhimurium, Campylobacter*, and *S. mutans*, have shown resistance to the antimicrobial action of FA (Luise et al., 2020). In the present study, we have evaluated the impact of FA on the clinical isolate of *A. baumannii* and *K. pneumoniae*, both recognized to be major causes of hospital infections. As documented in several studies, it has been found that these bacteria are present in a variety of animal and plant feeds, particularly in resistant strains. It was shown that it is also possible for *A. baumannii* to enter the hospital environment *via* kitchens or food handed in by the visitors (Campos et al., 2019). According to the findings of several investigations, *Acinetobacter* species may be found both in the kitchens of hospitals and in commonly consumed foods (Malta et al., 2020). Food-borne infections caused by these microbes are under-researched owing to the challenges in isolation, as well as in establishing the source of infection. Due to their tolerance to carbapenems and colistin and the possibility that these pathogens might spread resistance genes to other bacteria, these species pose a severe public health threat. While there is no in-depth study to conclusively establish that these microbes cause disease when they are consumed in food (De Amorim and Nascimento, 2017), it is a critical need of the hour to investigate more into this issue. A detailed understanding of the potential of these pathogens to induce VBNC by widely used food preservatives is also of paramount importance.

Carbapenem-resistant *K. pneumoniae* (CRKPE) strains have spread over the globe rampantly in the last decade (Cooke et al., 1980). Pneumonia, circulatory diseases, and sepsis in neonates and patients in critical care units are caused by *K. pneumoniae*, which is a common hospital-acquired pathogen. Although *K. pneumoniae* is mostly linked to nosocomial diseases, food has also been described as a probable transmission source for the bacteria (Casewell and Phillips, 1978b). *K. pneumoniae* was also isolated from Ready to eat food, raw meat, and raw vegetables, in addition to fruit juice and other raw foods (Casewell and Phillips, 1978a). Studies reported that the food served at the hospital was tested to be having more than 10 microorganisms per gram (Cooke et al., 1980). This species was also found to be extensively dispersed in the surroundings of the kitchen, which was thought to be the origin of the bacteria in food, at least to some extent (Ayliffe et al., 1970).

For this reason, it is of utmost importance to investigate various types of food additives for their safety and ulterior potential to induce VBNC and drive the emergence of AMR. In the present study, we have investigated the popular food additive FA for the same. The regulation allows the use of FA in various foods (milk, yogurt, cheeses, honey, wine fruit, and coffee) by direct use as a food additive or as a food preservative (FDA GRAS 688). Other than direct uses, it is also permitted as a constitute of paper and paperboard packaging material as an indirect list of food substances (FDA Code of Federal Regulation, 1976). Other than that, FA is also permitted to be used in animal drinking water and feed, in the form of sodium formate in dairy and soya products (FDA GRAS 688). In this study, we have reported that after FA treatment in most of food processing conditions, bacteria lose their culturability but are still metabolically viable on the basis of ATP production, membrane integrity, and respiratory activity. In the present study, flow cytometry data of CTC stained cells show that all three study isolates have a significant viable proportion of cells even after 10 days of FA treatment. All three species lose culturability after 4 days of FA treatment (Figures 1, 2 and Supplementary Figures 1, 2). This was also observed in the case of other similar food preservatives (Ogane et al., 2019). The FA treatment induces bacteria into entering a non-culturable state at temperatures ranging from 4 to 37^°^C. *E. coli* was earlier demonstrated to enter into the VBNC state at low temperature (Wei and Zhao, 2018), high-pressure CO_2_ (HPCD), and at suboptimal and optimal temperatures (Robben et al., 2018). In our study, the non-culturable bacteria in the VBNC state were still producing ATP, which was significantly higher than that observed in the non-viable control (Figures 1, 2 and Supplementary Figures 1, 2). After 10 days of treatment, when FA was removed, the increased ATP level corroborates with the previous report on increased ATP production after treatment removal (Robben et al., 2018, Robben et al., 2019). Gradual increase in ATP production with increasing incubation time after FA removal validated the VBNC state in *A. baumannii* (Figure 2), *K. pneumoniae*, and *E. coli* (Supplementary Figure 2). In addition to ATP production, membrane integrity and respiration rate of the non-growing cells were also estimated. Viable bacteria after FA treatment at low to optimal temperature maintain their membrane integrity and also show respiration ability. On the basis of membrane integrity and respiration ability, the viability percentage of non-growing bacteria was calculated. About 20–50% of non-growing bacteria still retained intact cell membrane and respiration activity (Figure 3 and Supplementary Figure 3). This finding is aligned with the previous report on membrane integrity in VBNC (Liu et al., 2018c). By employing CLSM and SEM, significant morphological variations in the exponential-phase cells, VBNC cells, and resuscitated cells were observed earlier (Wei and Zhao, 2018). In the present study, the morphology of VBNC and resuscitated cells was investigated in comparison to actively growing bacteria. It was observed that cells in the VBNC state were considerably smaller in size than the exponential-phase untreated cells. Furthermore, the majority of cells underwent a morphological transformation from rods to shorter rods or cocci shape. The average diameter of resuscitated cells was smaller than the exponential-phase cells but bigger than VBNCs (Figure 4 and Supplementary Figures 4–6). This finding is consistent with other studies using different physical and chemical agents for VBNC induction (Wei and Zhao, 2018). So, it is largely evident that the change in cell morphology during VBNC induction is xenobiotic-independent.

In this study, we have successfully resuscitated the VBNC bacteria by using fresh media and spent media (cell-free supernatant of growing culture) (Figure 5), which supports the previous evidence from the VBNC state of *Vibrio cholerae* induced by adding media from the growing culture of the same isolate (FernÁndez-Delgado et al., 2015). Cell-free supernatant of the growing culture supports the recovery of non-culturable bacteria faster than the fresh media (Kaprelyants et al., 1994; Bovill and Mackey, 1997; Pascoe et al., 2014), which was also reflected in the present study. Further studies exploring the role of the specific component of cell-free supernatant of the same species in enhancing the growth behavior of VBNCs may provide insights into the mechanism.

Due to reduced metabolic activities and cellular alterations in the VBNC state, these cells have enhanced antimicrobial resistance (Pienaar et al., 2016). Their high tolerance allows VBNC cells to sustain antimicrobial treatments in food and pharmaceutical settings, enabling further resuscitation, and probable recontamination of the surrounding environment. In terms of virulence and resistance patterns, earlier a close relationship was found between the *K. pneumoniae* isolate from the retail meat product and UTI isolates (Davis et al., 2015). In Patil et al. (2021) reviewed that biofilm formation by ESKAPE pathogens can be a potential source of food contamination and the presence of AMR and MDR genes poses an additional risk. Some reports have evidenced that VBNC bacteria resume their growth when they pass through the digestive tract in animals (Colwell et al., 1985; Linder and Oliver, 1989), as well as in human cell lines (Colwell et al., 1996). If MDR-resistant bacteria become non-culturable during food processing and storage, resuscitation of these bacteria after entering the host can act as a hidden risk to human health. This necessitates more elaborate studies on the risk of infection through VBNC in food for humans.

Because of its low metabolic rate, VBNC bacteria can sustain higher antibiotic pressure and are difficult to kill. After resuscitation, it may cause fatal diseases (Domingue et al., 1995; Mulvey et al., 2001; Rivers and Steck, 2001). Increased hygromycin and doxycycline resistance was observed in the non-culturable state of *Mycobacterium smegmatis* (Kusters et al., 2006). *Campylobacter jejuni* was resistant to chlorine-based disinfectants in the VBNC state (Rowe et al., 1997). Cold storage induced VBNC in *Vibrio vulnificus*, and prolonged cold incubation increased the resistance of VBNC cells to sonication, ethanol, and mechanical stress (Weichart and Kjelleberg, 1996). The MIC of vancomycin increased 500 times in the VBNC state of *Enterococcus faecalis* (del Mar, Lleò et al., 2007). Once these bacteria are resuscitated, they can cause fatal disease, as evident by the reports of reinfection or chronic ear infection by *Haemophilus influenza* and mice digestive tract infection by *H. pylori* (Ehrlich et al., 2002; She et al., 2003). In addition, bacteria may acquire an increased AMR pattern during VBNC induction that reflects after recovery from VBNC, as shown in the food isolate of *Listeria monocytogenes* (Olaimat et al., 2018). Environmental stress other than antibiotics triggers environmental adaptive stress management and AMR *via* altered expression of efflux pump in *L. monocytogenes* isolated from food (Verraes et al., 2013; Olaimat et al., 2018). Giacometti et al. (2021) comprehensively reviewed the role of xenobiotics other than antibiotics in the emergence of multidrug resistance. In most cases, efflux pumps play a major role in the emergence of MDR (Giacometti et al., 2021). In the present study, we observed that the AMR pattern in *A. baumannii* changed from sensitive to resistant for AMC-30, AMP, CFS50/50, CTX30, ES, GEN 50, and LE5. In the case of *K. pneumoniae*, the acquired AMR is for AMP-10, AT-30, CTX30, CD2, NIT200, RIF-5, TGC-15, and CFS50/50. *E. coli* susceptibility changed to resistant against E5, TOB10, MRP10, CIP5, and Gen50 after resuscitation (Figure 6). Like other organic acids, FA also exerts a broad effect on microbes, so it was hypothesized that FA may finally trigger the expression of outer membrane porins and antibiotic efflux pumps. Altered regulation of efflux pumps has earlier been shown to be responsible for multidrug resistance (Li and Nikaido, 2009; Nikaido and Pagès, 2012; Soto, 2013). To confirm this finding, we quantitated the expression of 10 different porins and efflux pump genes. Earlier global gene and proteome expression revealed that outer membrane proteins are involved in the evolution of bacteria in MDR (Peng et al., 2005; Alice et al., 2008). Both OmpH and OmpA are molecular markers for virulence also (Confer and Ayalew, 2013; Khamesipour et al., 2014). ABC transporter MacAB is involved in bacterial resistance against multiple antibiotics, antimicrobial peptides, and non-antibiotic xenobiotics and chemicals (arsenite) (Shi et al., 2019; Shirshikova et al., 2021). ABC transporter genes were also shown to be upregulated in VBNC induction (Liu et al., 2016). These were upregulated in VBNC and resuscitated bacteria in the present study. Efflux pumps EmrA and EmrB are responsible for the efflux of multiple antibiotics and co-resistance to heavy metals (Zhang et al., 2016; Lin et al., 2017). ABC transporter is responsible for iron acquisition through FusBC and regulator of fusD, and is involved in AMR (O’Neill and Chopra, 2006; Fernandes, 2016; Mosbahi et al., 2018). In the present study, we found up to a 10-fold increased expression of all these efflux pumps and porin genes in the VBNC and the resuscitated state (Figure 7). This increased expression of relevant efflux pumps and porins known to be responsible for causing AMR might be responsible for the increased resistance pattern after FA treatment.

Bacteria maintained constant and in some cases upregulated expression of virulence genes even in the non-culturable state (Jiang et al., 2021). Although due to the low metabolic rate in VBNC, bacteria are not able to initiate disease, but once these bacteria enter the host, their virulence can cause disease after resuscitation *in vivo* (Oliver and Bockian, 1995; Sun et al., 2008). In the present study, we examined the virulence of *K. pneumoniae* and *A. baumannii* strains using five different genes. The genes in *A. baumannii* were tonB, sidero, fimD, pilM, and lysE, while the genes associated with *K. pneumoniae* were IutA, fimH, fimD, entB, and pilA. In *A. baumannii*, VBNC and resuscitated bacteria at 4 and 37^°^C showed upregulation of all the above-mentioned virulence genes, whereas VBNC state at 25^°^C showed constant expression levels. *K. pneumoniae* also showed a similar pattern. Mutation in TonB and siderophores were earlier shown to reduce bacterial survival inside the host by affecting systematic dissemination and tissue localization (Khamesipour et al., 2014; Holden et al., 2016; Mosbahi et al., 2018). FimH, FimD, PilA, and PilM are usually involved in pili-mediated bacterial binding, invasion, and biofilm formation (Rosen et al., 2008; Sarshar et al., 2020). The expression of FimH, FimD, PilA, and PilM was elevated in the VBNC state and after resuming growth for only 37^°^C condition (Figure 8), whereas in other conditions, they maintained similar expression levels. Based on earlier evidence (Wu et al., 2021), a high prevalence of IutA and entB after treatment in our study may putatively make bacteria hypervirulent (Figure 8). The presence of pathogenic bacteria in a non-detectable state and the constant expression of virulence genes act as a significant hidden threat to human health. Furthermore, to evaluate the impact of VBNC cells inside the host cells to elucidate the pathogenicity of VBNC bacteria, *in vivo* studies are warranted and are a part of the future plan.

According to these findings, the existence of low quantities of food preservatives in an environment conducive to food manufacturing may function as a possible trigger for the development of AMR and induction into the VBNC state. Also, because of their need for survival, cells may attain irregular shape, thick cytoplasm, the gap between the cell walls, and reduced density in the nuclear area, and a dramatic shift in the intracellular organization may also be spotted (Robben et al., 2018). Based on previous findings and the results of our present study, there is a severe health concern posed by VBNC cells existing in food-processing facilities associated with healthcare settings. Because of the risk of resuscitation, spread, resistance to antibiotics, and ability to escape regular culture-based detection methods, VBNCs induced by food additives are a critical threat in this era of AMR being the next biggest pandemic. An elaborate study of the mechanism involved in VBNC induction and resuscitation-associated changes at the genomic, transcriptomic, and metabolomic levels is absolutely necessary and in progress, which is a part of our future study.

## Conclusion

To the best of our knowledge, this is the first report on integrating food processing and packaging hazards with hospital-associated pathogenic bacteria. Till date, VBNC in the food industry has only been explored with regard to food-borne pathogens and not hospital infection-associated ESKAPE pathogens. The outcome of our study definitively demonstrates that these pathogenic bacteria can enter the VBNC state after exposure to FA under standard food processing conditions, can be resuscitated easily, and can increase virulence and trigger new AMR traits after resuscitation. Resuscitated bacteria flared with significantly increased resistance against common antibiotics compared to the initial untreated actively growing bacteria. This article presents insightful research to demonstrate how a widely used and hitherto known to be safe food preservative poses a covert risk of inducing VBNC and AMR adversely affecting human health.

## Data availability statement

The datasets presented in this study can be found in online repositories. The names of the repository/repositories and accession number(s) can be found in the article/Supplementary material.

## Author contributions

SC conceived the study plan, designed the experiments, analyzed the data, and drafted the manuscript. MY, SD, PJ, and SA performed the experiments, analyzed the data, and contributed to drafting the manuscript. ST, VG, and DR performed the experiments and validations. All authors read and approved the final version of the manuscript.
