# Supplementary material for: Formic acid, an organic acid food preservative, induces viable-but-non-culturable state, and triggers new Antimicrobial Resistance traits in Acinetobacter baumannii and Klebsiella pneumoniae
Source: Front Microbiol. 2022 Nov 24;13:966207. doi: 10.3389/fmicb.2022.966207 (PMC9730046; doi:10.3389/fmicb.2022.966207)
Supplement: Supplementary file 1 [file Data_Sheet_1.docx]

**Supplementary figure legend**

**Figure-S1**

**VBNC optimization** VBNC induction after FA treatment was checked at day 2, day 4 and day 10. VBNC induction was confirmed by using two properties of VBNC state – non-culturability and metabolic energy production, in *K. pneumoniae* and *E. coli* with formic acid treatment at three different incubation temperatures, 4°C, 25°C, and 37°C. VBNC induction optimization in *K. pneumoniae* and *E. coli* (A & D) culturability assessment, (B & E) quantitative representation of culturability and (C & F) ATP production. All three study isolates show growth at day 2 but loss of culturability at day 4 to day 10 (A). This reduction of growth in VBNC was highly significant as compared to untreated growing bacteria from the same incubation temperature while non-significant to non-viable bacteria (B &E). Non-growing bacteria at day 4 and day 10 showed significant ATP production as compared to non-viable bacteria in all three study isolates (C &D).

FA- formic acid, GC- growing control, NVC- non viable control, 4C- sample incubated at 4°C, 25C- sample incubated at 25°C, 37C- sample incubated at 37°C, D_2- sample tested after 2 days of FA treatment, D_4- sample tested after 4 days of FA treatment, D_10- sample tested after 10 days of FA treatment; CFU colony formic unit, RLU- relative light unit, KP- *Klebsiella pneumoniae,* EC- *E. coli*.

**Figure-S2**

**VBNC validation** After VBNC induction further it was confirmed at day 10. VBNC induction by culturability and ATP production up to 4 hours after FA removal and addition of fresh media. Further viability percentage was checked on the basis of membrane integrity for all the study isolates and all three incubation temperatures. VBNC induction confirmation in *K. pneumoniae* (A, B, C, D) and *E. coli* (E, F, G & H) culturability detection (A & E), quantitative representation of culturability (B &F) and ATP production (C & G), PMA PCR assay (D & H). FA induced VBNC at all three-incubation temperatures did not resume detectable growth up to 4 hours after removal of FA and incubation in optimal growth conditions. After removal of FA, higher ATP levels indicated de novo metabolic energy production in non-culturable state (C & G). PMA PCR results showed up to 45% population maintained intact cell membrane in VBNC state (D & H). FA treatment in combination with 4°C incubation induced more VBNC as compared to 25°C and 37°C. While viability percentage of 37°C incubation was significant as compared to non-viable control.

FA- formic acid, GC- growing control, NVC- non viable control, 4C- sample incubated at 4°C, 25C- sample incubated at 25°C, 37C- sample incubated at 37°C, D_2- sample tested after 2 days of FA treatment, D_4- sample tested after 4 days of FA treatment, D_10- sample tested after 10 days of FA treatment; CFU colony formic unit, RLU- relative light unit*,* KP- *Klebsiella pneumoniae,* EC- *E. coli*.

**Figure-S3**

**Determination of respiratory activity as a viability marker after FA treatment.**  After VBNC confirmation further respiratory activity of bacteria at VBNC state was measured by the flow cytometry at day 2 and day 4 in *Acinetobacter baumannii* (A)*,* *Klebsiella pneumoniae* (B)*,* and *E. coli* *(*C) at incubation temperatures 4°C, 25°C and 37°C.

FA- formic acid, GC- growing control, NVC- non viable control, 4C- sample incubated at 4°C, 25C- sample incubated at 25°C, 37C- sample incubated at 37°C, D_2- sample tested after 2 days of FA treatment, D_4- sample tested after 4 days of FA treatment, D_10- sample tested after 10 days of FA treatment, AB- *Acinetobacter baumannii,* KP- *Klebsiella pneumoniae,* EC- *E. coli*, growing – actively growing culture from mid log phage, 4°C_GC- 4°C culture without treatment, 25°C_GC- 25°C culture without treatment, 37°C_GC- 37°C culture without treatment, 4°C_FA- 4°C culture with treatment, 25°C_FA- 25°C culture with treatment, 37°C_FA- 37°C culture with treatment.

**Figure- S4**

**Morphological changes of VBNC and resuscitation state after 4 days treatment.**  Morphology of VBNC and resuscitated sate in terms of shape and size was compared with growing bacteria under confocal laser scanning microscopy (CLSM) in *A. baumannii* in different growth conditions at 4°C (A, D)*,* at 25°C (B, E) and at 37°C (C, F). FA induced VBNC after 4 days treatment (A, B, C) and resuscitated (D, E, F).

**Figure -S5**

**Morphological changes of VBNC and resuscitation state in *K. pneumonia*.**  Morphology of VBNC and resuscitated sate in terms of shape and size was compared with growing bacteria under confocal laser scanning microscopy (CLSM) in *A. baumannii* in different growth condition at 4°C (A, B, C, D, E)*,* at 25°C (F, G, H, I, J) and at 37°C (K, L, M, N, O) (for *K. pneumoniae,* and *E. coli* refer supplementary figure S5 & S6). Untreated (A, F and K), day 4 VBNC (B, G and L), day 4 resuscitated (C, H and M), day 10 VBNC (D, I and N) and day 10 resuscitated (E, J and O).

**Figure -S6**

**Morphological changes of VBNC and resuscitation state in *E. coli.***  Morphology of VBNC and resuscitated sate in terms of shape and size was compared with growing bacteria under confocal laser scanning microscopy (CLSM) in *A. baumannii* in different growth condition at 4°C (A, B, C, D, E)*,* at 25°C (F, G, H, I, J) and at 37°C (K, L, M, N, O) (for *K. pneumoniae,* and *E. coli* refer supplementary figure S5 & S6). Untreated (A, F and K), day 4 VBNC (B, G and L), day 4 resuscitated (C, H and M), day 10 VBNC (D, I and N) and day 10 resuscitated (E, J and O).
