# Supplementary material for: Formic acid, an organic acid food preservative, induces viable-but-non-culturable state, and triggers new Antimicrobial Resistance traits in Acinetobacter baumannii and Klebsiella pneumoniae
Source: Front Microbiol. 2022 Nov 24;13:966207. doi: 10.3389/fmicb.2022.966207 (PMC9730046; doi:10.3389/fmicb.2022.966207)
Supplement: Supplementary file 2 [file Data_Sheet_2.docx]

Supplementary Table-S1 : AMR and virulence gene specific primers of *Acinetobacter baumannii* and *Klebsiella pneumoniae*

| **Gene Name** | **Gene Role** | **Primer Sequence** |  |  |
| --- | --- | --- | --- | --- |
| *Acinetobacter baumannii* |  | |  |  |
| OmpA | AMR | F’- cttctatgcaactttggac  R’- ggttagaagaaccataacct |  |  |
| OmpH | AMR | F’ - ttgcgacaacaggcacttac  R’ - gttggctctggcattttgat |  |  |
| MdtA | AMR | F’ - cttaccgattatgcttactg  R’ - tagaggtaacgtacaagctc |  |  |
| MacA | AMR | F’ - cgaccacccaagaaaacagt  R’ - tgaacgcgttatttttgcag |  |  |
| MacB | AMR | F’ - accatgaacagtgacagtat  R’ - gtacaccaccaattaaacac |  |  |
| FusD | AMR | F’ - atgggtgagtttaatgtcta  R’ - aaggtaaaaacacaaattga |  |  |
| FusBC | AMR | F’- gggtatggtgtcgtctaag  R’- aggagtccgatctaataaag |  |  |
| EmrA | AMR | F’- ctttaaactctcaagtggtg  R’- agttcttctttagacacagc |  |  |
| EmrB | AMR | F’- attagtgacaacctgtcttg  R’- ccacagaattaacagaccta |  | F’- attagtgacaacctgtcttg  R’- ccacagaattaacagaccta |
| abeM | AMR | F’- gaggtatgcaagatactcag  R’- ggtattcaaatagagtcgtg |  |  |
| fimH | Virulence | F’ - aatataacggcacctcttac  R’ – gagtcgctattgtagttgtt |  |  |
| fimD | Virulence | F’ - ggtgcgttttctctataac  R’ – ccagattgtagtagtcggta |  |  |
| entB | Virulence | F’ – ggcaatccctaaattacag  R’ – atattagccaccactttctc |  |  |
| pilA | Virulence | F’ - cagacctttgtcccttac  R’ - gttcagactctcctgacc |  |  |
| 16srRNA | Housekeeping gene | F’ - actcctacgggaggcagcag  R’ - ggcgtggactaccagggtatc |  |  |
| ***Klebsiella pneumoniae*** |  |  |  |  |
| OmpA | AMR | F’ – gcattggttactacatgg  R’ - ctcatactcttttagcacca |  |  |
| OmpH | AMR | F’ – gagcggattatacctatacc  R’ - gtctgatacgtgacatcct |  |  |
| MdtA | AMR | F’ - acgatgagaataaggtcag  R’ - gtgacaacttctaccttcg |  |  |
| MacA | AMR | F’- taaactgaaagacattctgc  R’- tagtaatcacgttcttcacc |  |  |
| MacB | AMR | F’ - gtgtatcctggtaaggattt  R’ - atgccgtagacattaaaata |  |  |
| FusD | AMR | F’ - gtcttgttgggtttctttat  R’ - agggagagatccattagtag |  |  |
| FusBC | AMR | F’ - ttttcaatgataatttgagc  R’ - attcaaattcatgctcact |  |  |
| EmrA | AMR | F’ - ctgacattgctcttcattat  R’ - ggtcttagccttttcaaac |  |  |
| EmrB | AMR | F’ - ggctatatcagcgataacta  R’ - cagaatgataatctcactgg |  |  |
| abeM | AMR | F’ - ttttatctatggtcatttcg  R’ - aaacagagtgacttcaaaga |  |  |
| iutA | Virulence | F’ - gagtcagcaggtatttgac  R’ – gacctttgtactcatacagc |  |  |
| fimH | Virulence | F’ - aatataacggcacctcttac  R’ – gagtcgctattgtagttgtt |  |  |
| fimD | Virulence | F’ - ggtgcgttttctctataac  R’ – ccagattgtagtagtcggta |  |  |
| entB | Virulence | F’ – ggcaatccctaaattacag  R’ – atattagccaccactttctc |  |  |
| pilA | Virulence | F’ - cagacctttgtcccttac  R’ - gttcagactctcctgacc |  |  |
| 16srRNA | Housekeeping gene | F’ - actcctacgggaggcagcag  R’ - ggcgtggactaccagggtatc |  |  |

Supplementary Table S2 : Antibiotics used for antimicrobial susceptibility test

| **S. No.** | **Antibiotics name** | **Abbreviation** | **Concentration** |
| --- | --- | --- | --- |
| 1 | Amikacin | AK30 | 30 mcg/disc |
| 2 | Amoxiclav (Amoxicillin/ Clavulanic acid) | AMC30 | 30 mcg/disc |
| 3 | Ampicillin | AMP10 | 10 mcg/disc |
| 4 | Aztreonam | AT30 | 30 mcg/disc |
| 5 | Cefepime | CPM50 | 50 mcg/disc |
| 6 | Cefixime | CFM5 | 5 mcg/disc |
| 7 | Cefoperazone (Sulbactum) | CFS 50/50 | 50/50 mcg/disc |
| 8 | Cefotaxime | C10 | 10 mcg/disc |
| 9 | Cefoxitin/ Cloxacillin | CXX 30/200 | 30/200 mcg/disc |
| 10 | Cefpodoxime | CPD10 | 10 mcg/disc |
| 11 | Ceftazidime | CAZ30 | 30 mcg/disc |
| 12 | Chloramphenicol | CTX30 | 30 mcg/disc |
| 13 | Ciprofloxacin | CIP5 | 5 mcg/disc |
| 14 | Clindamycin | CD2 | 2 mcg/disc |
| 15 | Co-Trimoxazole (sulpha/ Trimethoprim) | COT25 | 25 mcg/disc |
| 16 | Vancomycin | VA30 | 30 mcg/disc |
| 17 | Ertapenem | ETP10 | 10 mcg/disc |
| 18 | Erythromycin | E5 | 5 mcg/disc |
| 19 | Gentamycin | GEN50 | 50 mcg/disc |
| 20 | Levofloxacin | LE5 | 5 mcg/disc |
| 21 | Linezolid | LZ10 | 10 mcg/disc |
| 22 | Meropenem | MRP10 | 10 mcg/disc |
| 23 | Minocycline | MI30 | 30 mcg/disc |
| 24 | Nitrofurantoin | NIT200 | 200 mcg/disc |
| 25 | Rifampicin | RIF5 | 5 mcg/disc |
| 26 | Tazobactam/Piperacillin | PIT 100/10 | 100/10 mcg/disc |
| 27 | Teicoplanin | TE 10 | 10 mcg/disc |
| 28 | Tigecycline | TGC 15 | 15 mcg/disc |
| 29 | Tobramycin | TOB 10 | 10 mcg/disc |
| 30 | Tetracycline | TEI 30 | 30 mcg/disc |
