# Supplementary figures and images for: Formic acid, an organic acid food preservative, induces viable-but-non-culturable state, and triggers new Antimicrobial Resistance traits in Acinetobacter baumannii and Klebsiella pneumoniae
Source: Front Microbiol. 2022 Nov 24;13:966207. doi: 10.3389/fmicb.2022.966207 (PMC9730046; doi:10.3389/fmicb.2022.966207)

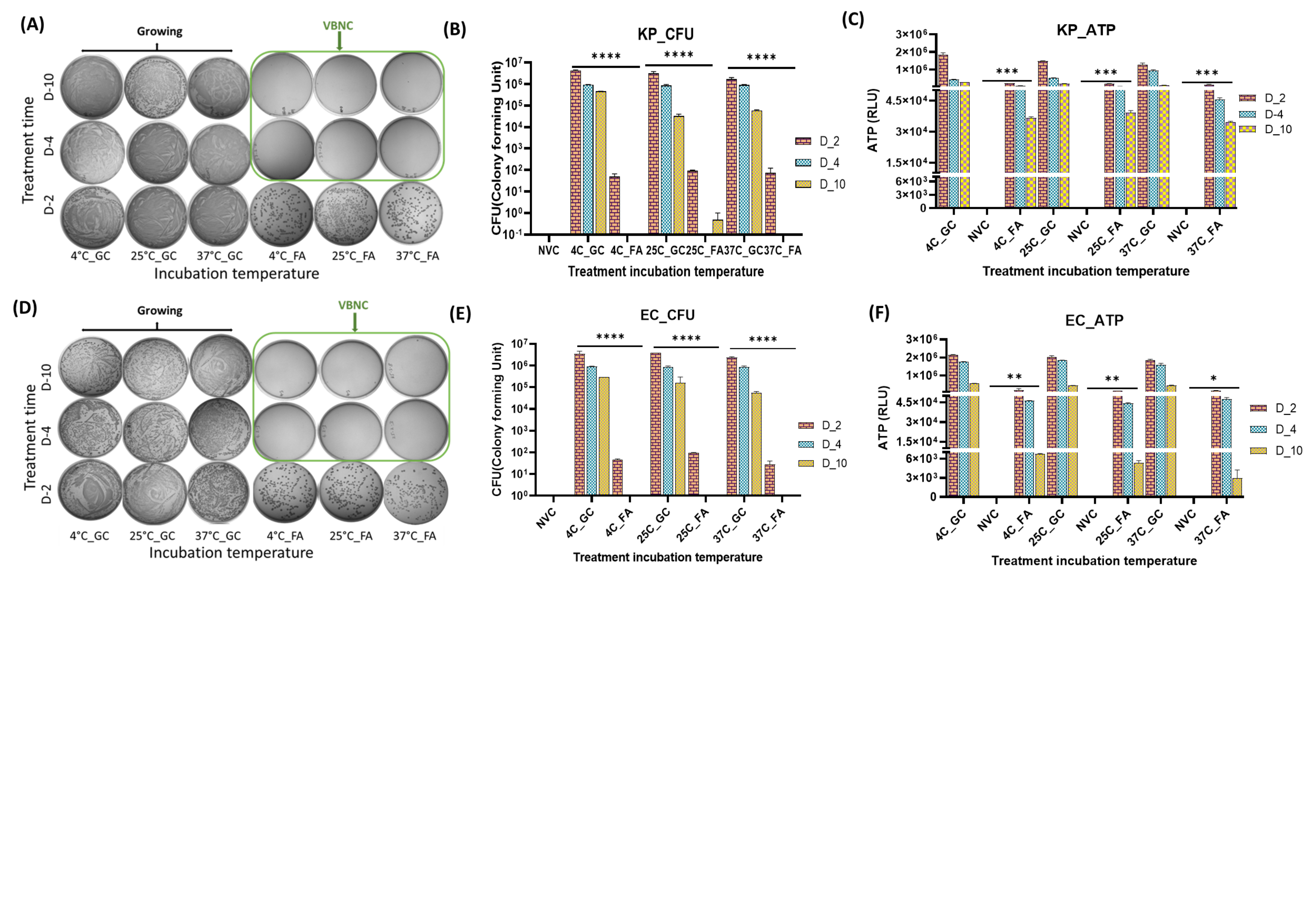

Supplement: Supplementary file 3 [file Image_1.JPEG]

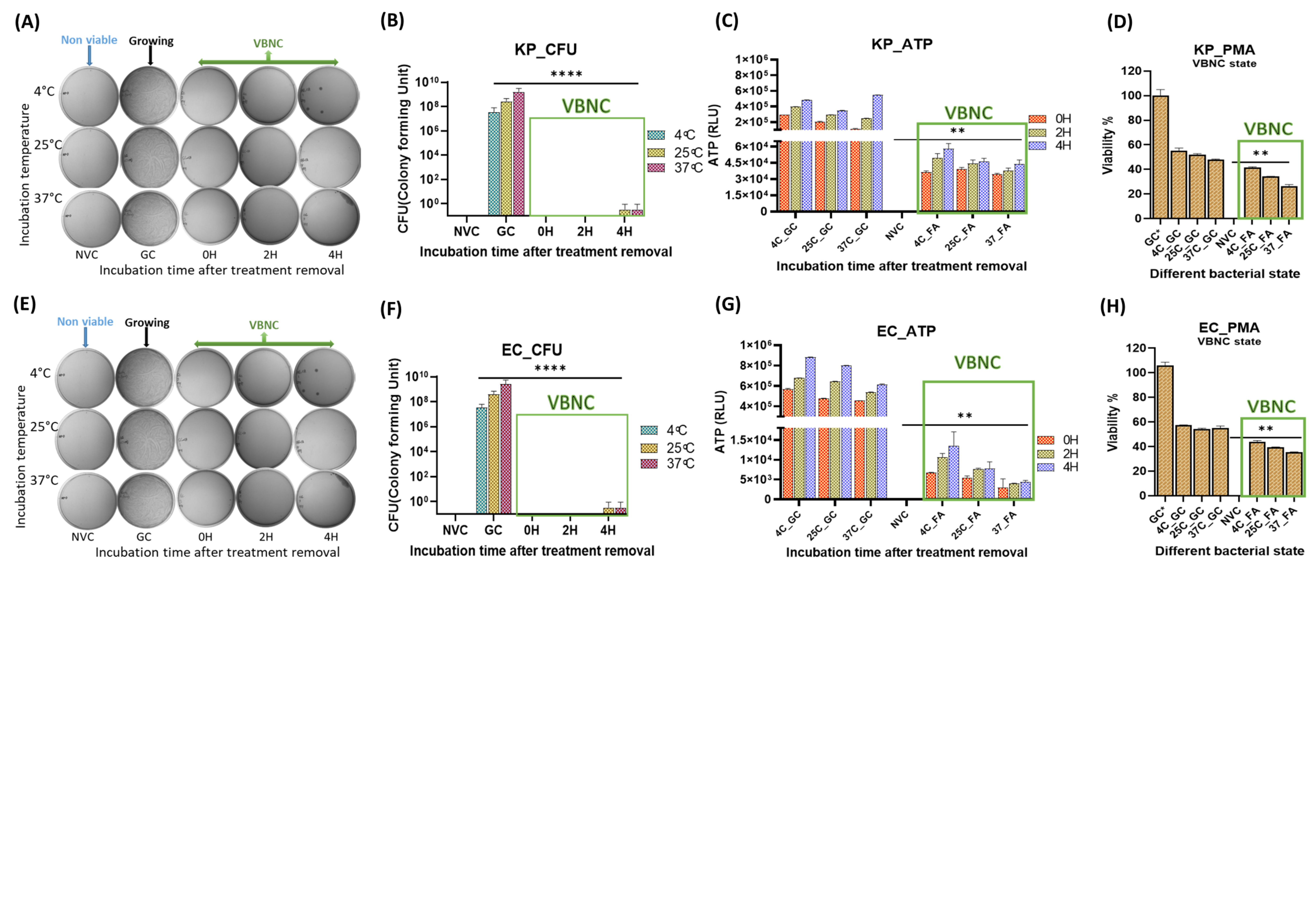

Supplement: Supplementary file 4 [file Image_2.JPEG]

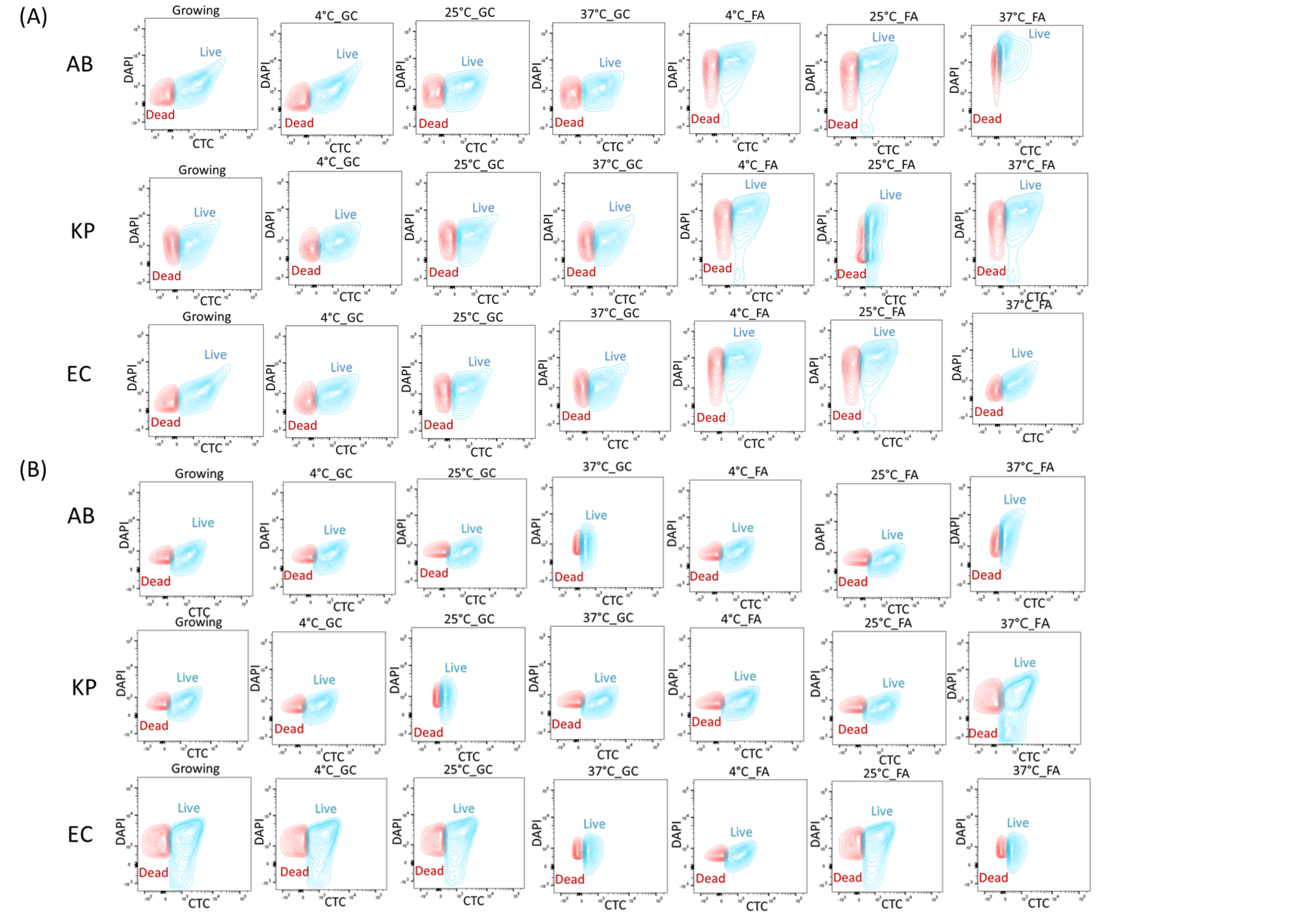

Supplement: Supplementary file 5 [file Image_3.JPEG]

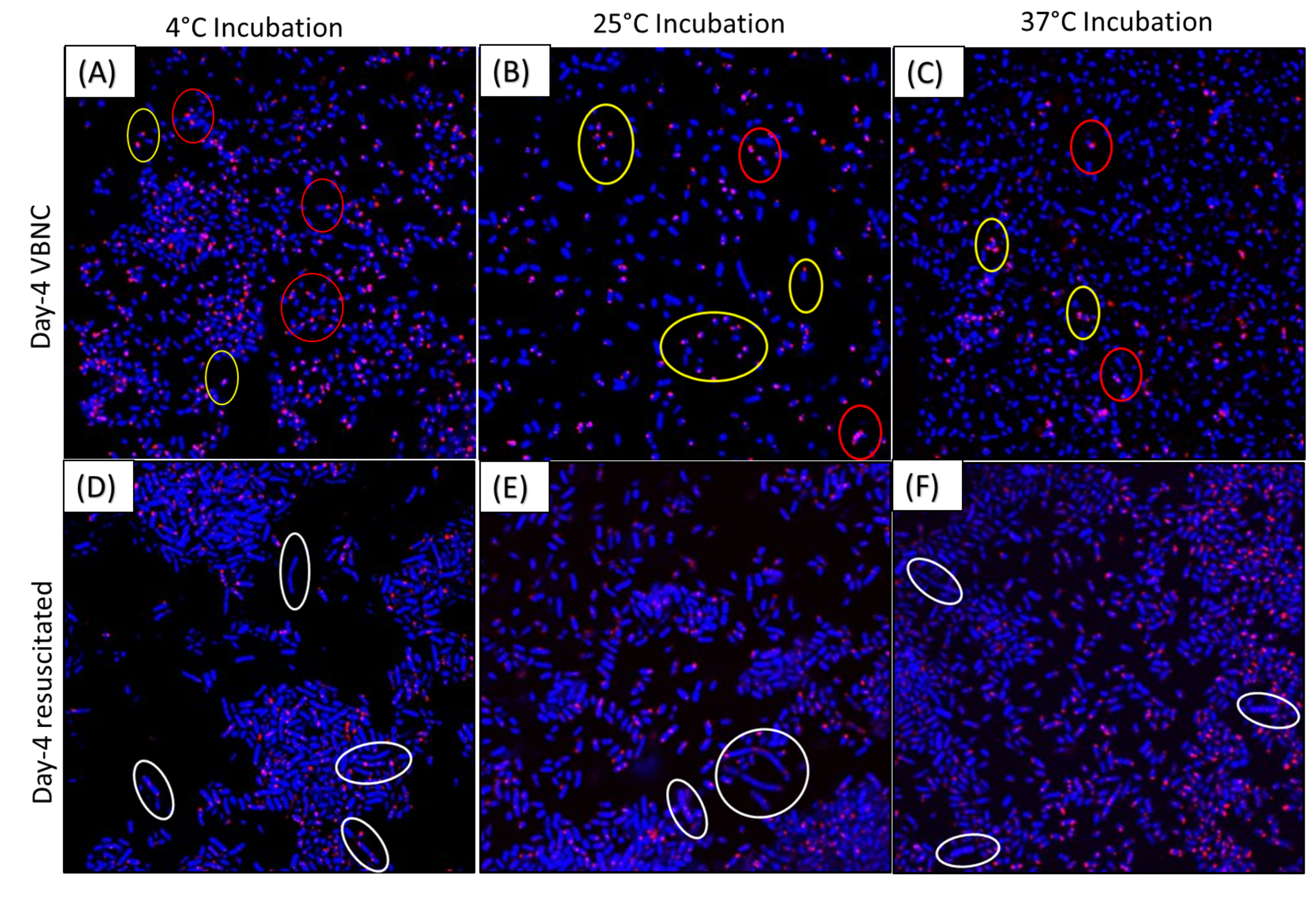

Supplement: Supplementary file 6 [file Image_4.JPEG]

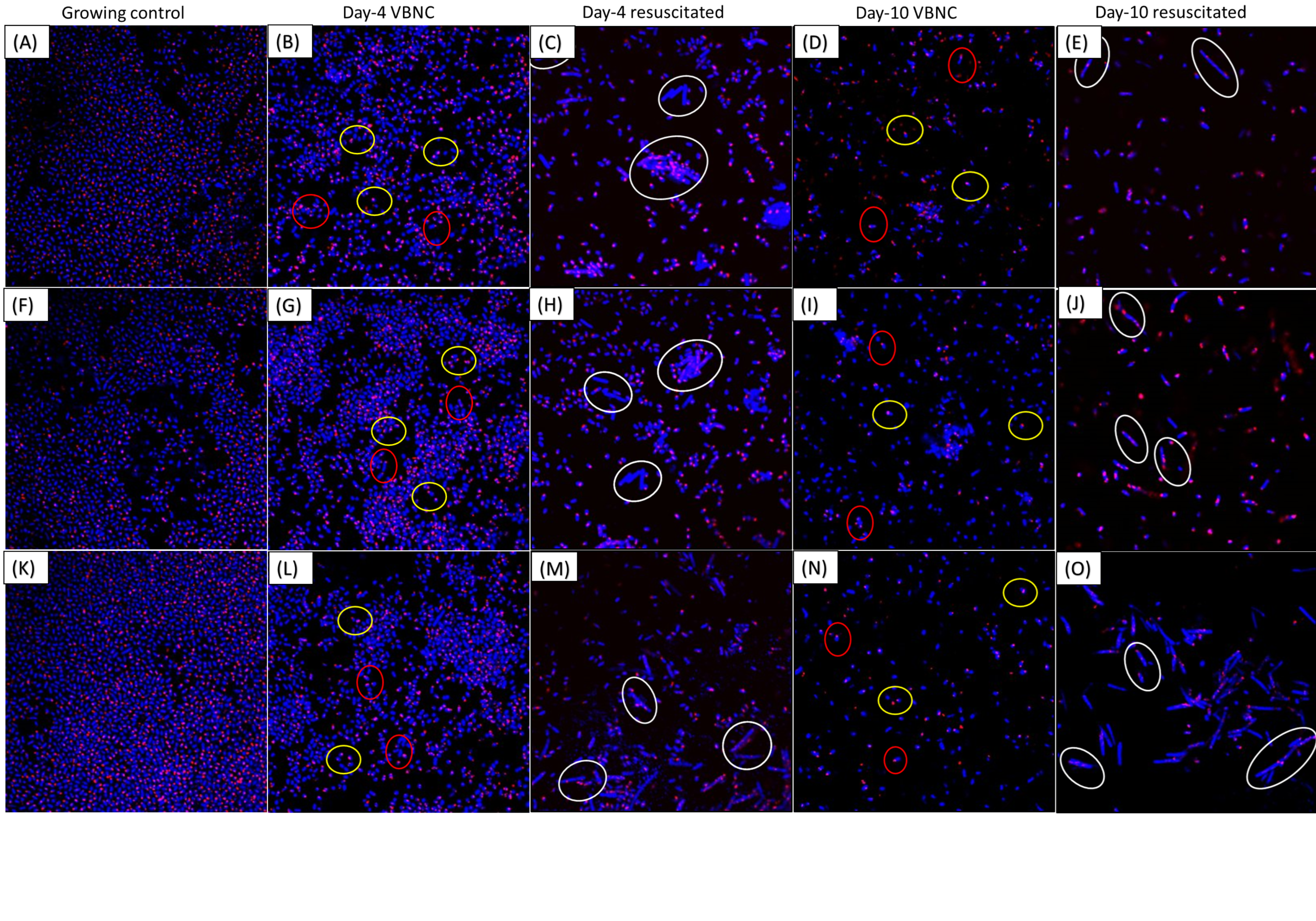

Supplement: Supplementary file 7 [file Image_5.PNG]

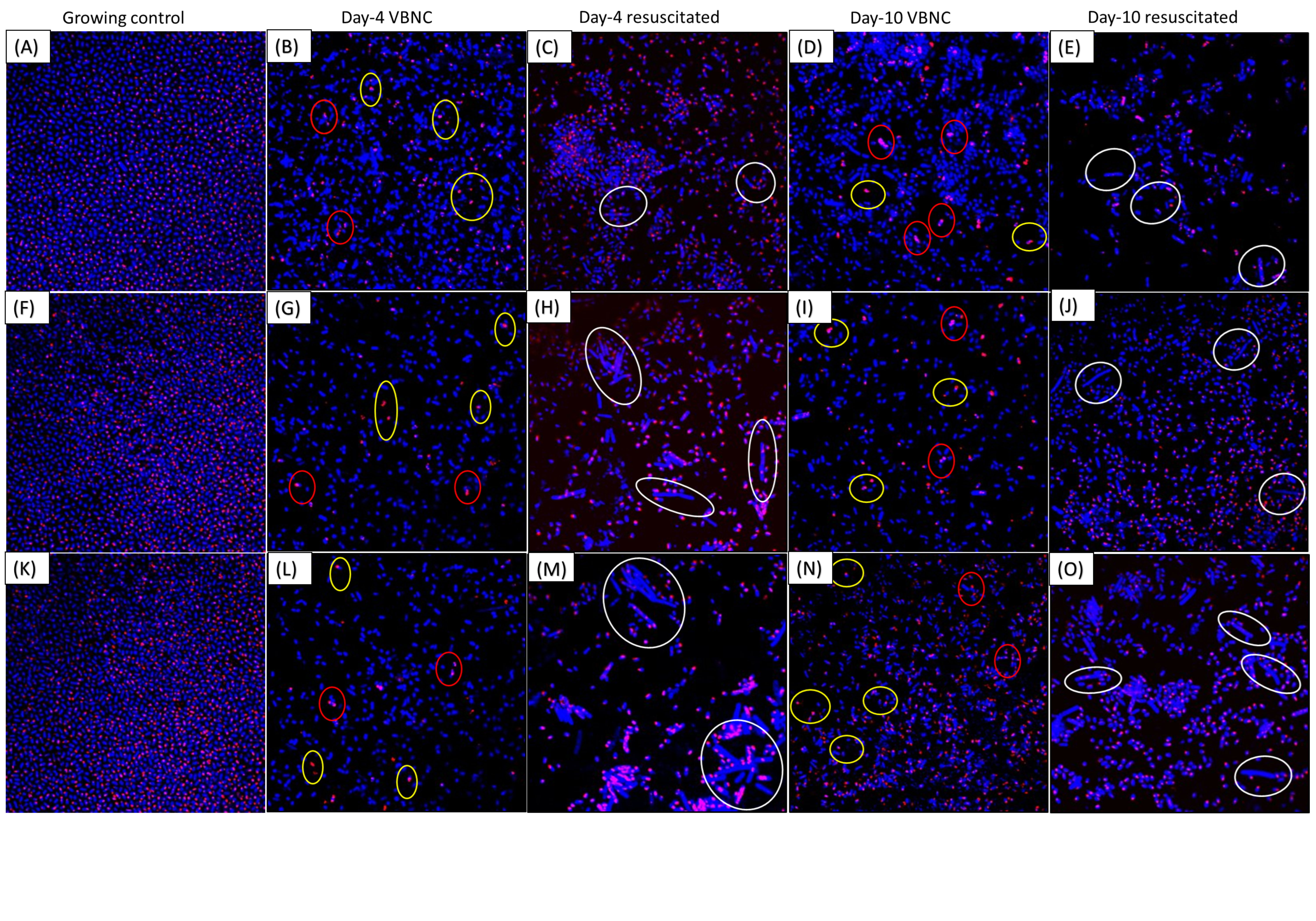

Supplement: Supplementary file 8 [file Image_6.JPEG]
